# Successful Management of Iatrogenic Aortocoronary Dissection Induced by a Guide Extension Catheter Using Intravascular Ultrasound Guidance

**DOI:** 10.7759/cureus.102741

**Published:** 2026-01-31

**Authors:** Yu Sugawara, Tamio Nakajima

**Affiliations:** 1 Cardiovascular Medicine, Yamato Kashihara Hospital, Kashihara, JPN

**Keywords:** complications, guide extension catheter, iatrogenic aortocoronary dissection, intravascular ultrasound, percutaneous coronary intervention

## Abstract

Iatrogenic aortocoronary dissection is an uncommon but potentially life‑threatening complication of percutaneous coronary intervention. We describe a case caused by a guide extension catheter, in which intravascular ultrasound (IVUS) enabled precise assessment and successful percutaneous management. A 79-year-old female with exertional chest pain underwent percutaneous coronary intervention after persistent symptoms despite optimal medical therapy. Severe, heavily calcified stenosis in the distal right coronary artery required balloon dilatation with a guide extension catheter. Following dilatation, a National Heart, Lung, and Blood Institute (NHLBI) type F dissection with contrast staining in the sinus of Valsalva was detected. The patient remained hemodynamically stable, and the guidewire was in the true lumen. IVUS identified the dissection entry point and extensive intramural hematoma. A drug‑eluting stent was deployed to seal the flap, restoring normal coronary flow. Post‑procedural coronary computed tomography confirmed that the dissection had not propagated into the ascending aorta and was completely sealed. Because the patient remained stable without ischemia or aortic regurgitation, conservative management was continued. Her recovery was uneventful, and follow‑up computed tomography at 10 months demonstrated complete healing. Although guide extension catheter-related aortocoronary dissection is rare, this case highlights the value of IVUS for defining the injury and guiding stent placement. Careful attention to catheter position and avoidance of automatic contrast injection are essential. Early recognition and prompt sealing of the entry site, followed by imaging to assess aortic involvement, are critical for optimal outcomes.

## Introduction

Aortocoronary dissection is a critical complication due to the restricted coronary flow, leading to cardiac ischemia. If the dissection spreads widely, it becomes fatal. It is mainly induced by the diagnostic catheter or guiding catheter tip [1]. When we perform percutaneous coronary intervention (PCI), we often use a guide extension catheter to enhance the backup support. Though a guide extension catheter is convenient, it occasionally injures the coronary artery intima [2], resulting in retrograde dissection [3]. Contrast injection after tip injury may worsen the situation [4] as local intimal injection increases intraluminal pressure. Intravascular ultrasound (IVUS) is a useful device for evaluating the injured coronary artery and detecting the dissection entry site [5]. Furthermore, it provides valuable information about the vessel diameter and extent of the hematoma. To address this critical complication, maintaining coronary flow before the occurrence of a circulatory collapse is essential [1]. In cases of iatrogenic coronary dissection, therapeutic decision‑making should be directed by the patient’s overall clinical status, including hemodynamic stability and the extent and severity of the dissection, as classified by the angiographic systems of the National Heart, Lung, and Blood Institute (NHLBI) and Dunning et al. [6,7]. The NHLBI system divides the dissection of the coronary artery into six types based on angiographic appearances of the intimal disruption and contrast clearance: type A dissection represents radiolucent areas within the coronary lumen during contrast injection, with minimal or no persistence of contrast; type B dissection represents parallel tracts or double lumen separated by radiolucent area during contrast injection, with minimal or no presence; type C dissection appears as contrast outside lumen with persistence of contrast in the area after clearance of contrast from the coronary lumen; type D dissection represents spiral luminal filling defects, frequently with extensive contrast staining of the vessel; type E dissection appear as new, persistent filling defects; and type F dissection represents those that lead to total occlusion of the coronary artery, without anterograde flow [6]. Dunning et al. proposed classifications based on the extent of the dissection. Class I is defined as focal dissection restricted to the coronary cusp, class II extends to the ascending aorta up to 40 mm, and class III extends to >40 mm from the ascending aorta [7]. Management options include conservative treatment, coronary stent placement, or aortic root repair, with or without coronary artery bypass surgery [8]; however, the best bail-out strategy is not fully understood. The incidence of iatrogenic aortocoronary dissection is in the range of 0.02-0.15% [8], with aortic involvement occurring in 0.021% of the cases [4]. Generally, this rare complication is caused primarily by diagnostic or guiding catheters. Moreover, when it occurs, there is usually insufficient time to assess the coronary artery with IVUS. In the present case, however, the aortocoronary dissection was induced by a guide extension catheter, and we were able to evaluate the injured artery using IVUS because the patient remained hemodynamically stable. The guide extension catheter-induced aortocoronary dissection was successfully treated with IVUS‑guided stent implantation; therefore, we report this case.

## Case presentation

**Figure 1:**
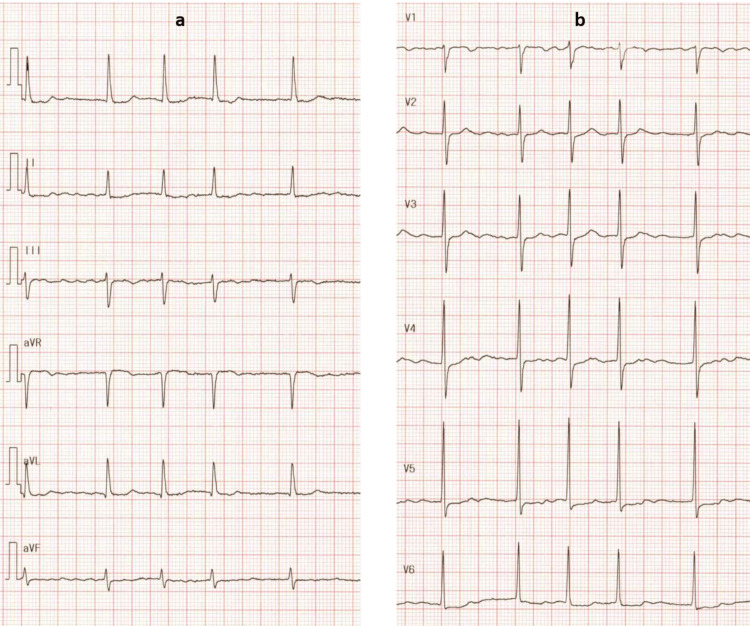
Electrocardiography showing atrial fibrillation. a: Limb leads demonstrating ST-segment depression in leads I, II, and aVL. b: Precordial leads demonstrating ST-segment depression in leads V3-V6.

A 79-year-old female with hypertension, hypercholesterolemia, cerebrovascular disease, and atrial fibrillation presented with exertional chest pain. Electrocardiography revealed atrial fibrillation with ST-segment depression in the limb leads I, II, and aVL (Figure 1A), as well as in the precordial leads V3-V6 (Figure 1B).

**Figure 2:**
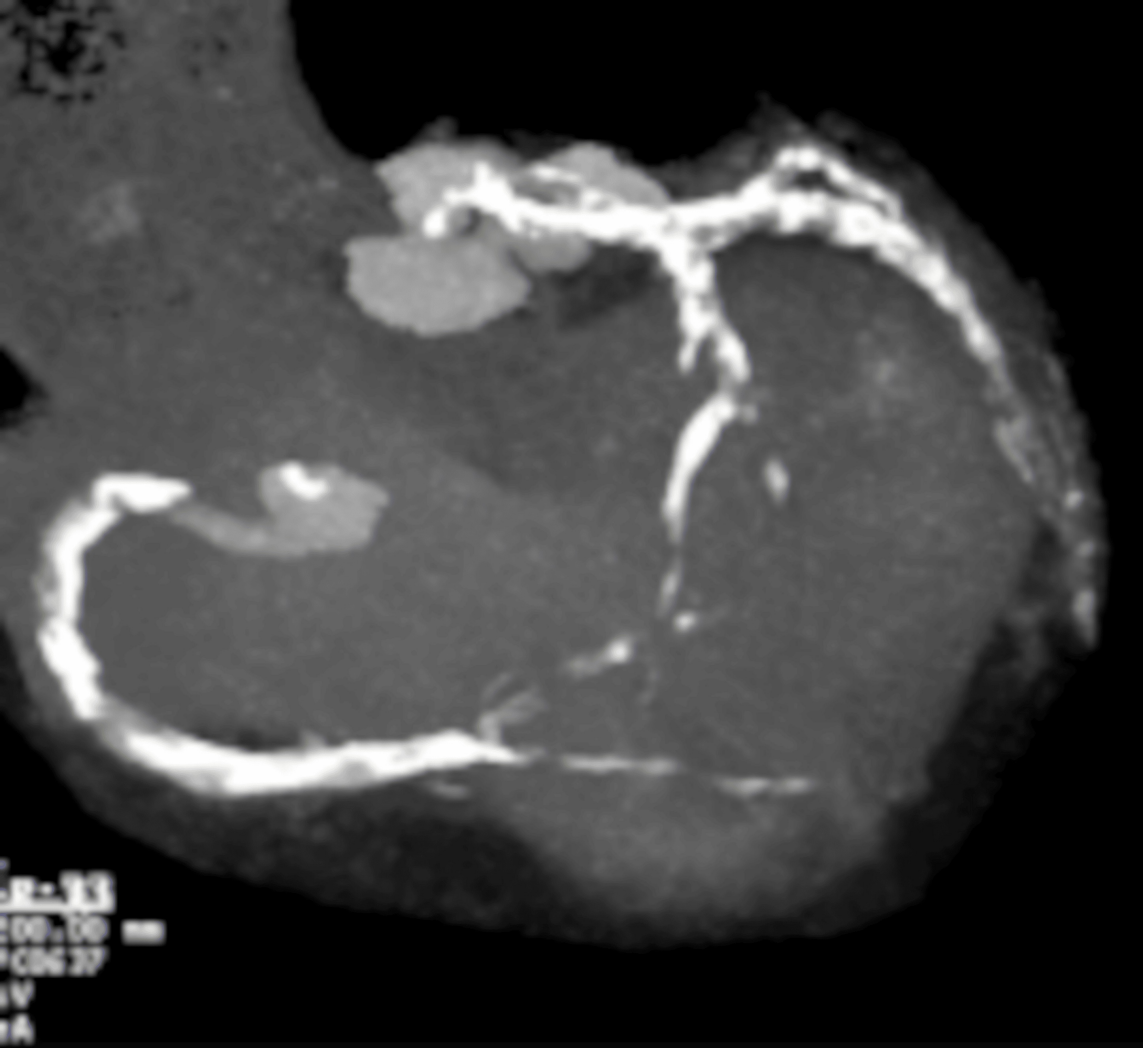
Coronary computed tomography angiography showing severe calcification in the right and left coronary arteries.

Echocardiography showed an ejection fraction of 53%, and left atrium dilatation with basal inferior wall motion hypokinesis. The low-density lipoprotein cholesterol level was 104 mg/dL. The patient was treated with amlodipine (5 mg), valsartan (80 mg), hydrochlorothiazide (12.5 mg), rosuvastatin calcium (2.5 mg), clopidogrel sulfate (75 mg), and rivaroxaban (10 mg). Despite optimal medical therapy, her symptoms persisted, and she underwent coronary computed tomography (CT) angiography, which revealed severe calcification in both coronary arteries (Figure 2).

**Figure 3:**
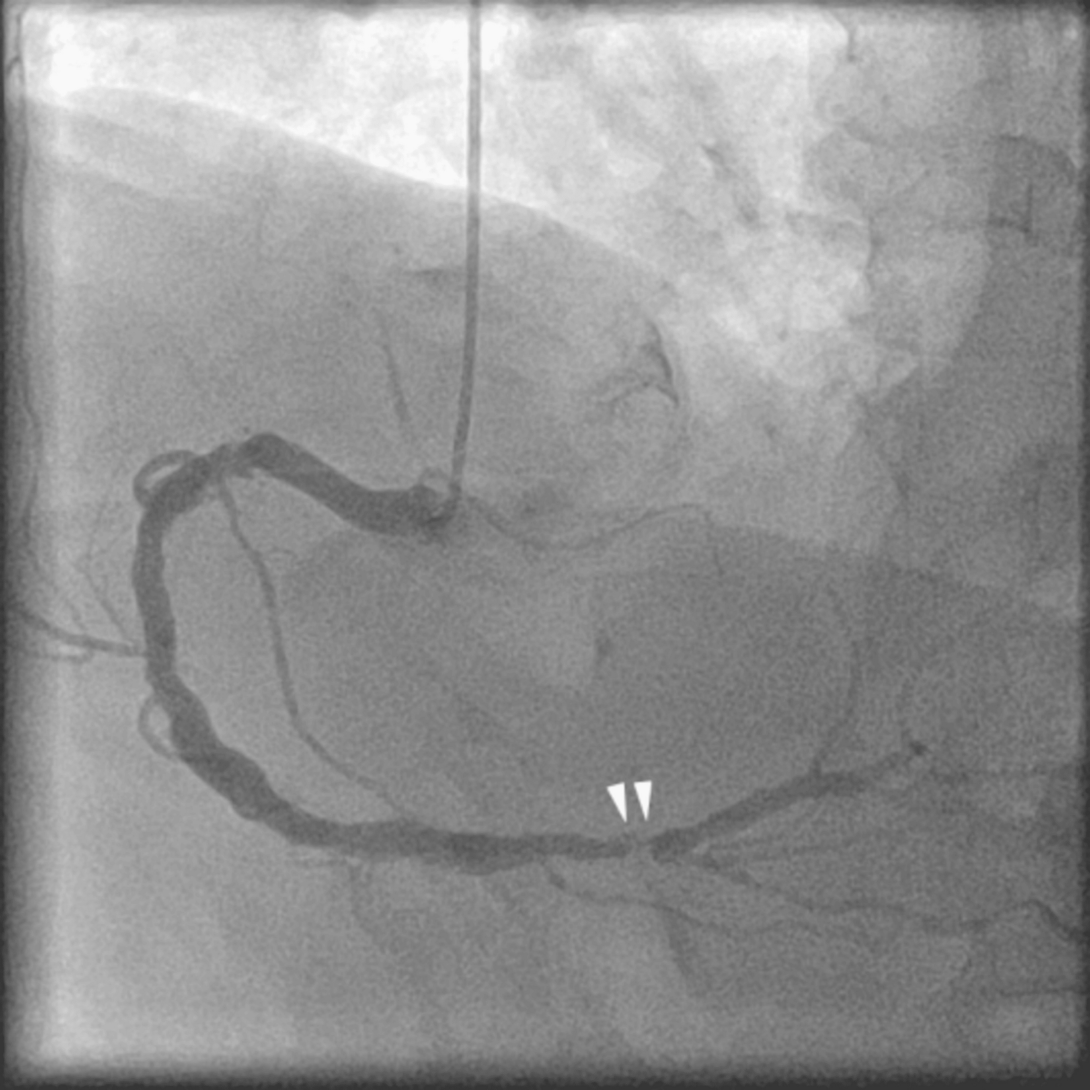
Right coronary angiography showing stenosis in the distal segment (white arrowheads).

**Figure 4:**
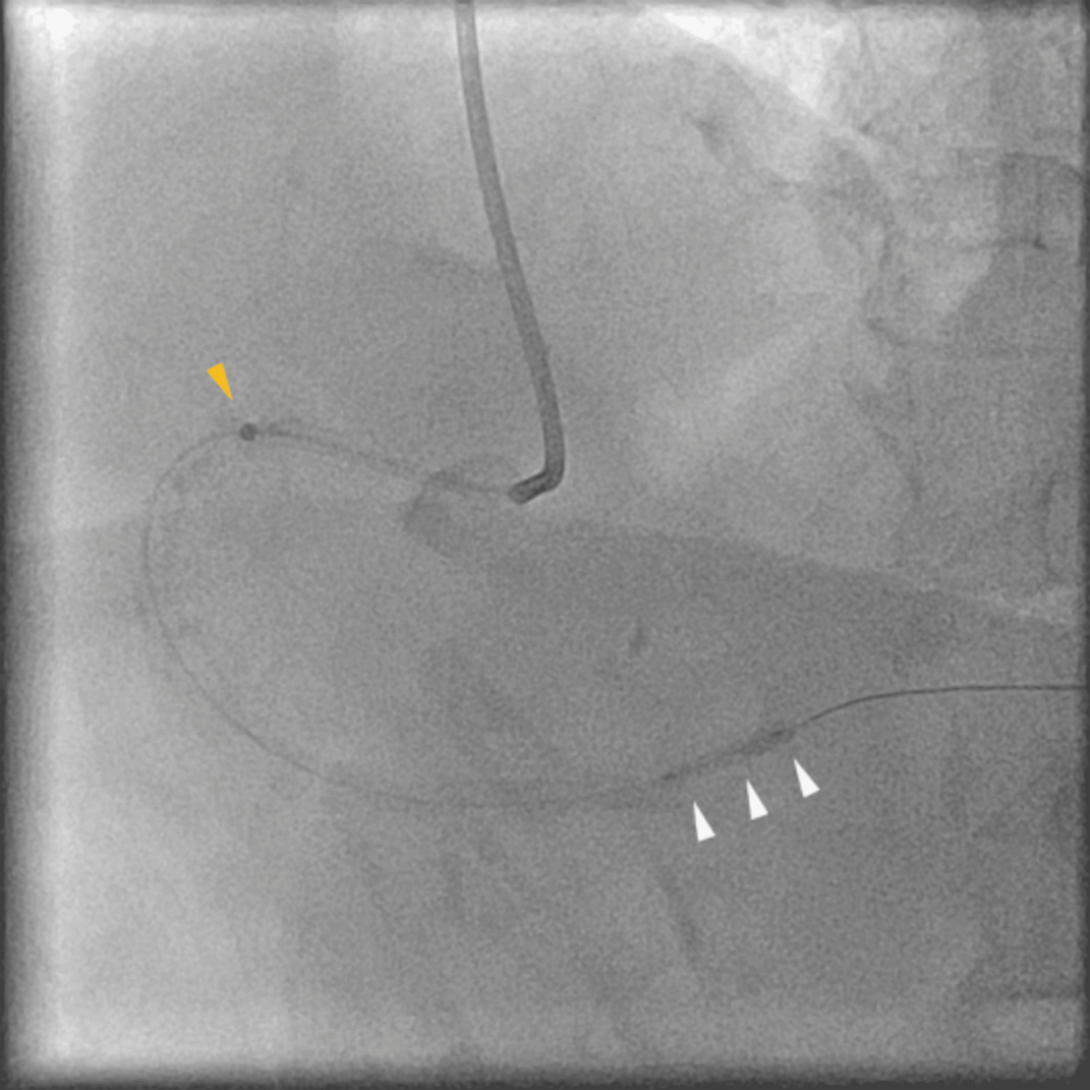
Balloon angioplasty with guide extension catheter support. For additional support, a guide extension catheter was advanced (orange arrowhead), and the lesion was subsequently dilated using a balloon (white arrowheads).

**Figure 5:**
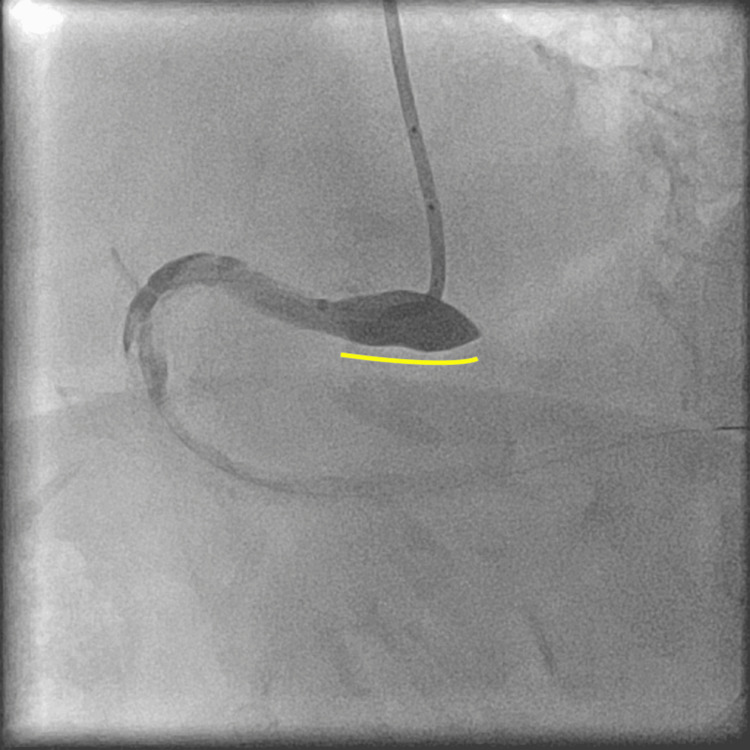
National Heart, Lung, and Blood Institute type F dissection, extending to the sinus of Valsalva. After plain old balloon angioplasty, a coronary angiography showed a National Heart, Lung, and Blood Institute type F dissection, extending to the sinus of Valsalva (yellow line).

The stenosis could not be clearly evaluated; thus, coronary angiography (CAG) was performed. It showed stenosis in the distal part of the right coronary artery (Figure 3, white arrowheads); therefore, PCI was performed. At that time, the patient’s blood pressure was 136/94 mmHg, and the heart rate was 88 beats per minute. We used a 6 F Mac1 Amplatz Left 0.75 guiding catheter (Boston Scientific, Marlborough, MA) and crossed the lesion with a 0.014-inch Sion blue guidewire (Asahi Intecc, Aichi, Japan). The guiding catheter was not engaged coaxially, and poor support impeded the smooth performance of PCI. The balloon or stent cannot cross the lesion; thus, we used a guide extension catheter (Hikyaku, Kaneka Medix, Osaka, Japan; Figure 4, orange arrowhead). After introducing the guide extension catheter, we performed IVUS (OptiCross, Boston Scientific, Marlborough, MA). Based on the IVUS findings, we dilated the lesion with a 3.0 x 12 mm balloon (Hiryu Plus, Terumo, Tokyo, Japan; Figure 4, white arrowheads) using inflation twice, at 12 atm for 15 seconds each, while the arterial pressure was normal. After scoring balloon angioplasty, CAG revealed NHLBI type F aortocoronary dissection extending to the sinus of Valsalva (Figure 5, yellow line, and Video 1).

**Video 1:** National Heart, Lung, and Blood Institute type F dissection, extending to the sinus of Valsalva.

**Figure 6:**
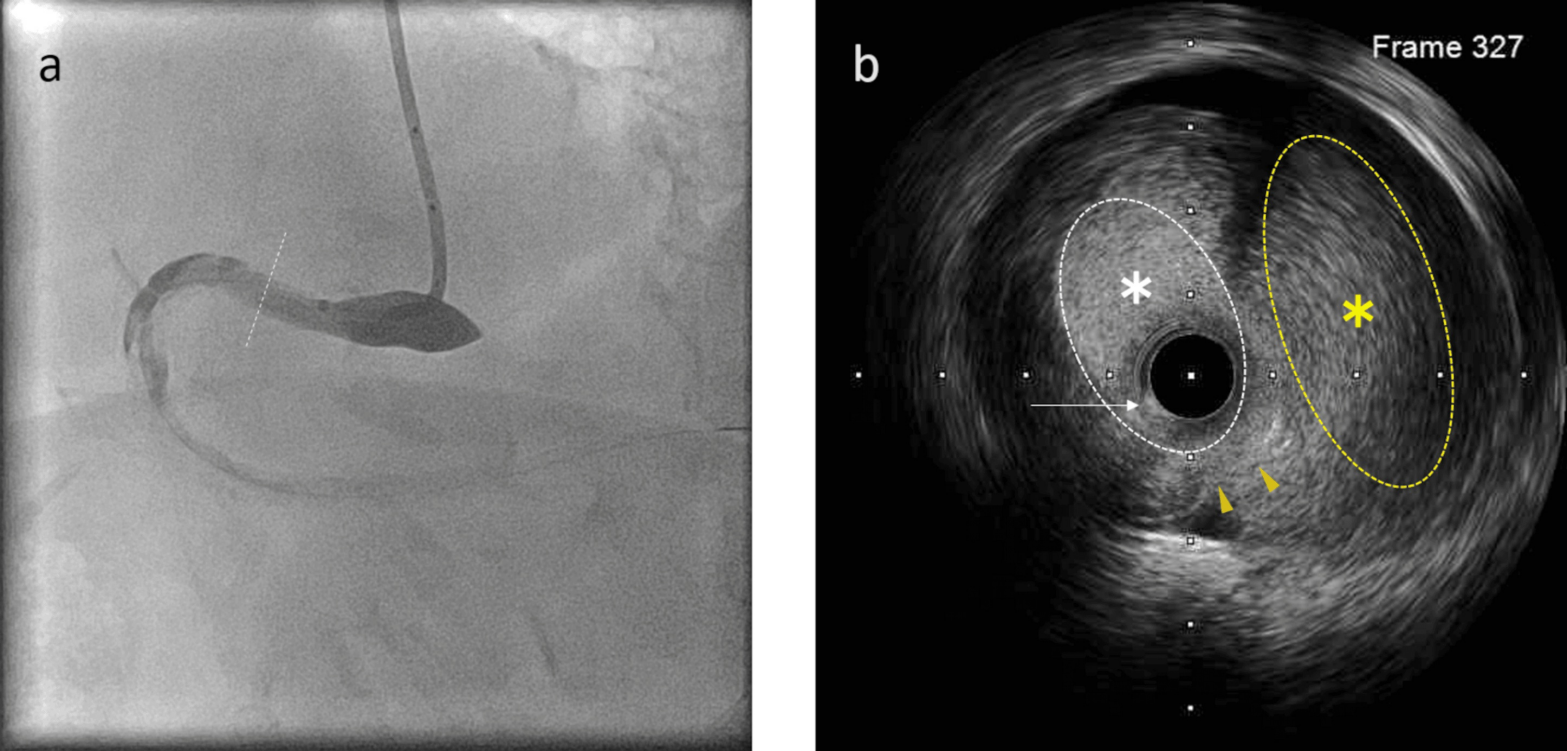
Right coronary angiography and cross-sectional intravascular ultrasound image. a: The white dotted line in the right coronary angiography indicates the location of the corresponding cross-sectional intravascular ultrasound image. b: Cross-sectional intravascular ultrasound image showing the true lumen (white asterisk) and pseudo-lumen (yellow asterisk). The guidewire is located in the true lumen (white arrow). Intimal dissection is also visible (orange arrowheads).

**Figure 7:**
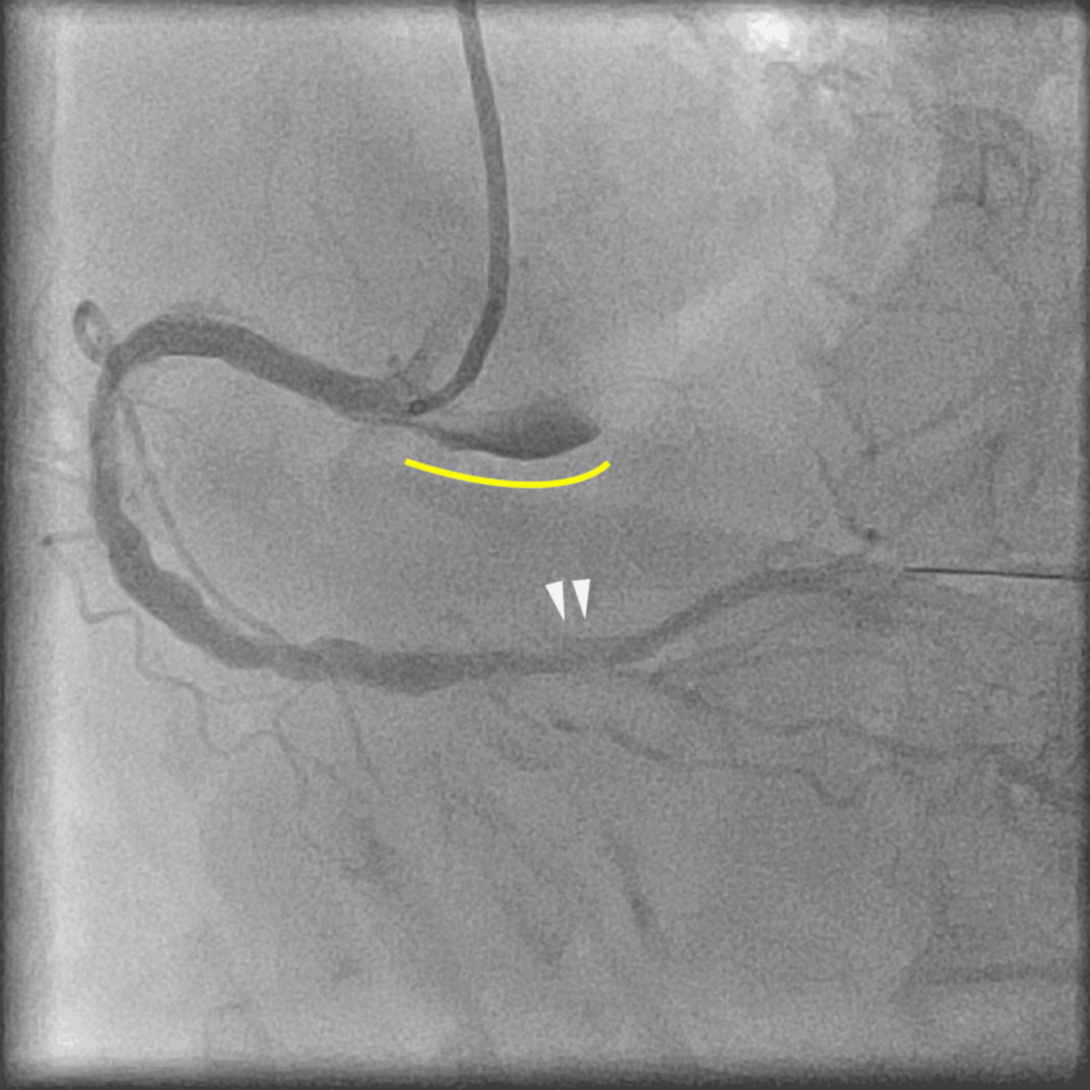
Final coronary angiography showing resolution of the dissection and recovery of antegrade coronary flow, although residual contrast staining remained in the sinus of Valsalva (yellow line). Dilation of the distal right coronary artery is noted (white arrowheads).

At that time, a guidewire was placed in the distal true lumen, the patient’s blood pressure was 135/54 mmHg, and the heart rate was 105 beats per minute. IVUS was then advanced approximately 2 mm distally to identify the entry site of the coronary dissection. IVUS showed a pseudo-lumen with intimal dissection (Figure 6B and Video 2), located approximately 10 mm from the ostium, along with an entirely intramural hematoma with coronary ostium involvement; therefore, we deployed a Xience Skypoint 4.0 x 28 mm stent (Abbott Laboratories, Chicago, Illinois), with three inflations at 12 atm for 10 seconds each, to treat the dissection and seal the flap under IVUS guidance. Post-dilatation was not performed. Final CAG demonstrated complete resolution of the dissection with restoration of antegrade coronary flow (Video 3); however, residual contrast staining was still present in the sinus of Valsalva (Figure 7, yellow line), and the target lesion had been dilated (Figure 7, white arrowheads).

**Video 2:** Intravascular ultrasound (IVUS) video demonstrating a National Heart, Lung, and Blood Institute type F coronary artery dissection.

**Video 3:** Final coronary angiography.

**Figure 8:**
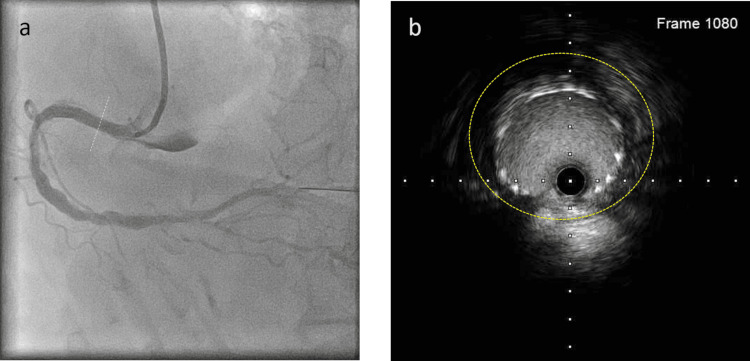
Final coronary angiography and cross-sectional intravascular ultrasound image. a: The white dotted line in the right coronary angiography indicates the location of the corresponding cross-sectional intravascular ultrasound image. b: Cross-sectional intravascular ultrasound image showing the stent was deployed roundly; however, a hematoma remained entirely around the stent (yellow dots).

Final IVUS demonstrated that the minimal cross-sectional stent area was 8.36 cm^2^, with a maximum diameter of 3.66 mm and a minimum diameter of 2.91 mm. At the dissection site, the stent cross-sectional area was 12.06 cm^2^, with a maximum diameter of 4.20 mm and a minimum diameter of 3.81 mm, and the hematoma remained entirely around the stent (Figure 8B and Video 4).

**Video 4:** Intravascular ultrasound after stent implantation.

The total contrast volume was 180 ml, the fluoroscopy time was 18.6 minutes, and the total fluoro exposure was 1,360 mGy. The total procedure time was 1 hour and 45 minutes.

**Figure 9:**
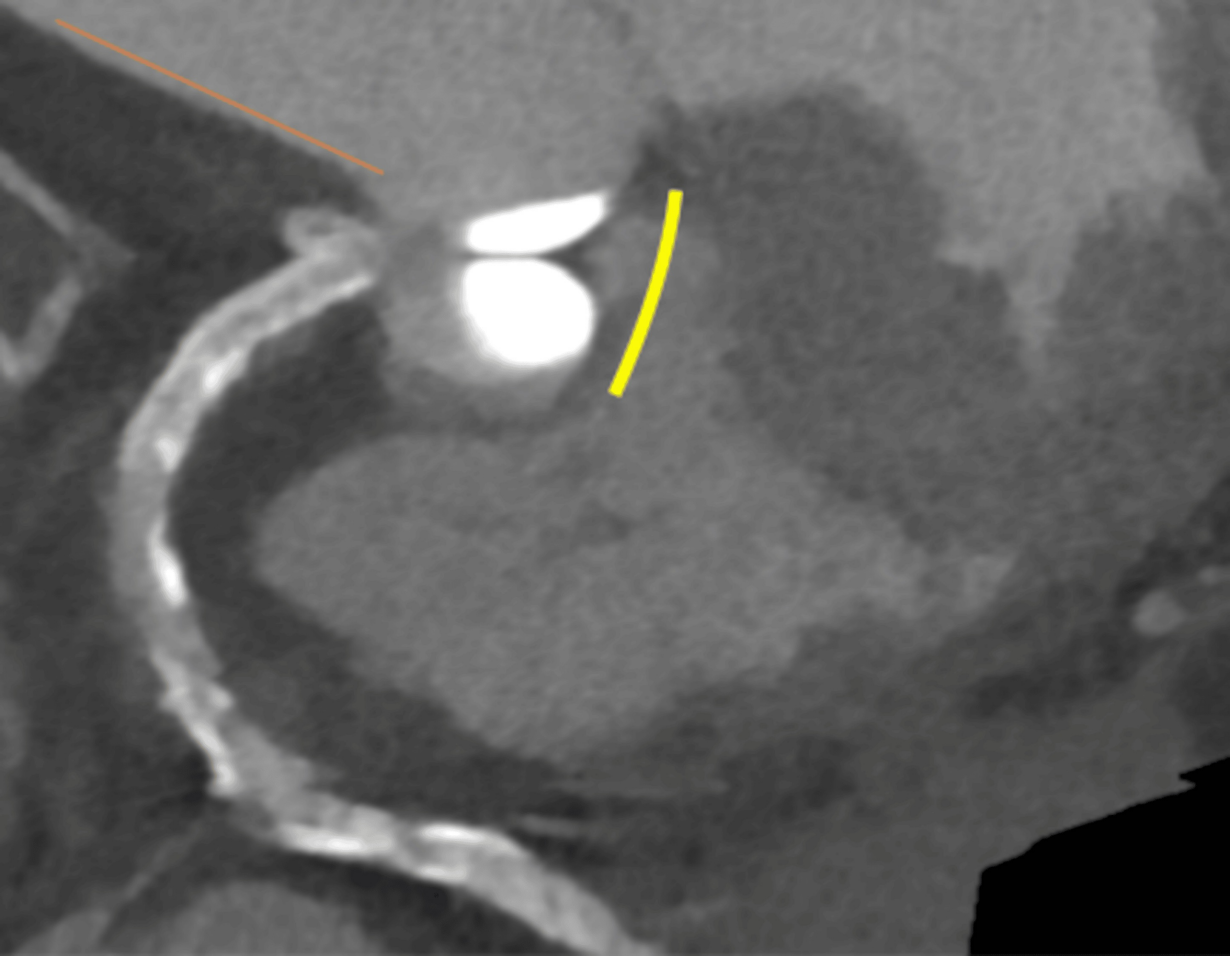
Postprocedural contrast-enhanced computed tomography. Axial contrast-enhanced CT imaging demonstrates retained contrast within the sinus of Valsalva (yellow line) without evidence of ascending aortic dissection (orange line).

We performed cardiac CT after PCI, which showed that the contrast remained in the sinus of Valsalva, but the dissection did not progress in the ascending aorta (Figure 9, yellow line and orange line).

**Figure 10:**
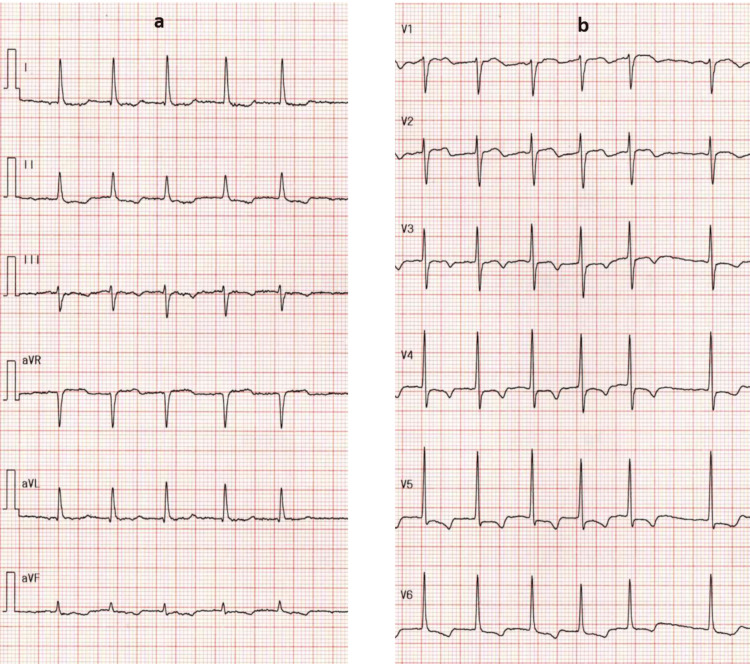
Electrocardiography one day after percutaneous coronary intervention. ECG showing atrial fibrillation. a: Limb leads demonstrating ST-segment depression in leads I, II, and aVF. b: Precordial leads demonstrating ST-segment elevation in V1 and V2, ST-segment depression in V4-V6, and T-wave inversion in V1-V6.

After careful observation, the patient did not experience chest pain, and at that time, her blood pressure was 121/81 mmHg, and her heart rate was 100 beats per minute. ECG one day after PCI revealed atrial fibrillation with the limb leads demonstrating ST-segment depression in leads I, II, and aVF, and the precordial leads demonstrating ST-segment elevation in V1 and V2; ST-segment depression in V4-V6; and T-wave inversion in V1-V6 (Figure 10).

**Figure 11:**
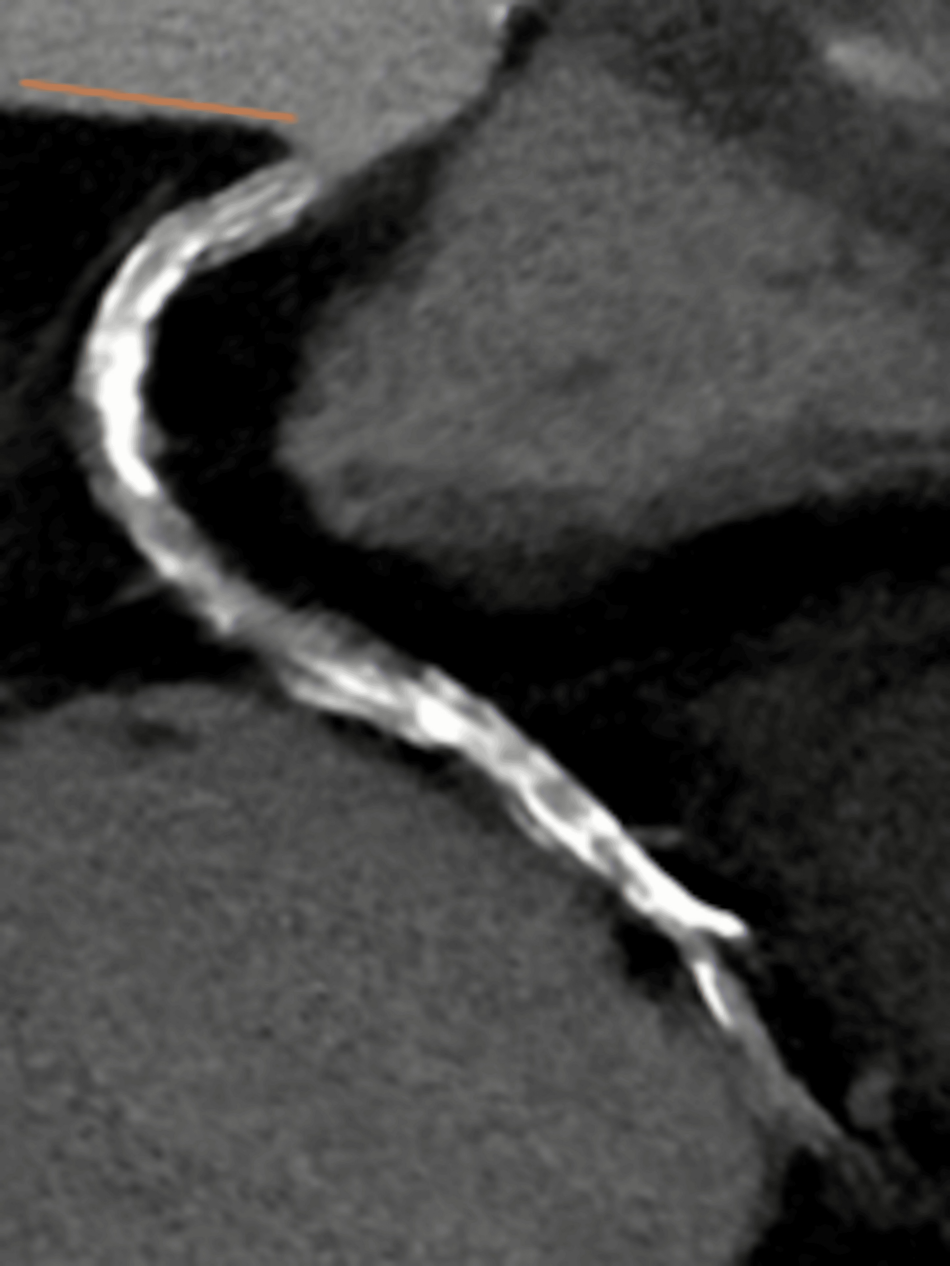
Axial contrast-enhanced computed tomography obtained 10 months after PCI. Axial contrast-enhanced CT obtained 10 months after percutaneous coronary intervention (PCI) shows no residual contrast within the sinus of Valsalva and no evidence of aortic dissection (orange line).

The maximum creatine phosphokinase level was 375 IU/L. The patient was discharged three days later. Aspirin was administered for approximately two weeks, after which it was discontinued. Ten months after PCI, contrast-enhanced CT demonstrated complete healing of the aortocoronary dissection (Figure 11, orange line), and the patient remained in good clinical condition.

## Discussion

The incidence of guiding catheter-induced iatrogenic aortocoronary dissection is rare (approximately 0.02-0.15%) [8]. Aortic involvement occurs in about 0.021% of the cases [4]. A previous report noted that the in-hospital mortality rate of iatrogenic coronary artery dissection with aortic dissection was 6.25% [4], and further mortality did not occur over a median follow-up period of 3.6 years [6]. When the dissection extends into the ascending aorta or involves the aortic valve, the condition becomes life‑threatening.

Iatrogenic coronary artery dissection begins with mechanical trauma caused by the catheter tip, and this expands circumferentially and longitudinally. As a result, coronary blood flow is restricted, which induces coronary ischemia [1]. Coronary arteries involving severe calcification are more likely to experience coronary dissection [9]. The guide extension catheter is frequently used during complex PCI to enhance the guiding catheter backup; however, it sometimes causes complications [2]. Both guiding catheter-induced aortocoronary dissection and guide extension catheter-induced dissection occur at the catheter tip, whereas guiding catheter-induced iatrogenic coronary artery dissection starts at the coronary ostium. The guide extension catheter-induced dissection we encountered involved intimal injury at a site remote from the ostium and subsequently propagated in a retrograde manner. In this case, the guide extension catheter was deep to obtain sufficient back-up. The right coronary artery ostium diameter was approximately 4.0 mm, the catheter outer size was approximately 2 mm, and the inner size was approximately 1.80 mm because we used a 6F guiding catheter. The outer diameter of the guide extension catheter was 1.75 mm, and the inner diameter was 1.48 mm. The diameter of the guide extension catheter was smaller than that of the right coronary artery, so we proceeded with the guide extension catheter. IVUS showed no calcification at the dissection site; however, the target coronary artery was overall heavily calcified.

There are three catheter-induced dissection mechanisms: wedged contrast injection, forceful catheter engagement, and deep catheter insertion [4]. Deep intubation or guide‑extension advancement can cause mechanical intimal injury if the tip catches on or tracks along the vessel wall. Repeated advancement and withdrawal can increase the opportunity of intimal disruption, especially in tortuous or calcified segments. Once the intima is compromised, even a routine contrast injection can propagate a dissection plane because contrast follows the path of least resistance. Real‑world dissections are often multifactorial with the interaction of vessel anatomy, catheter shape, support requirements, hemodynamics, and operator maneuvers. A previous case report showed iatrogenic aortocoronary dissection triggered by automatic contrast injection using a guide extension catheter support [10]. In this case, contrast injection during optical coherence tomography (OCT) triggered an adverse event. In our case, repeated advancement and withdrawal of the guide extension within a severely calcified coronary artery resulted in intimal injury, and subsequent contrast injection exacerbated the hematoma. Automatic contrast injection further worsened the situation because it raises the local pressure of the vessel. On the other hand, we can adjust the power and volume of contrast using manual injection. After we detected this complication by angiography, we avoided using a contrast injection to prevent further dissection. In the case of iatrogenic coronary artery dissection, the indication for the surgery is instability of hemodynamics, continuing ischemia, and aortic regurgitation due to increased aorta diameter [9]. In this case, the hemodynamics were stable, and no acute aortic regurgitation occurred; hence, we deployed the stent.

The classifications of aortocoronary dissection, the NHLBI, and Dunning classifications are common [7]. NHLBI types A and B are benign and are treated conservatively if the patient is hemodynamically and angiographically stable. However, NHLBI types C-F dissections potentially have the risk of acute closure; therefore, stent deployment is recommended [6]. Most Dunning class I patients are treated with a coronary stent or conservative therapy [8]. This was an NHLBI type F and Dunning class I case. Her blood pressure remained normal. The bailout strategy at this time was stent implantation because the patient was hemodynamically stable. Since the dissection was not spread at ascending aorta, first we implanted a coronary stent to restore the coronary flow, then we thought about the next strategy.

IVUS is a favorable device for assessing plaque morphology and coronary dissection [5,11]. In addition, it enables evaluation of stent expansion after deployment. When the dissection occurred, the patient remained hemodynamically stable; therefore, we performed IVUS to obtain maximal information regarding the injured artery to detect the dissection entry site and the lesion length. IVUS demonstrated a vessel diameter of 4.0 mm, an entry site located approximately 1.0 mm distal to the coronary ostium, and diffuse propagation of the hematoma. To ensure complete coverage of the dissection site, a 4.0 x 28 mm^2^ drug-eluting stent was selected. After stent placement, the dissection did not extend to the aorta with a favorable outcome. To evaluate the coronary artery, intracoronary imaging modalities such as IVUS and OCT are typically used. However, OCT was not employed in this case because it requires coronary flushing to clear erythrocytes, and such flushing can exacerbate the false lumen by enlarging the dissection site.

After PCI, a contrast-enhanced CT scan was performed to assess the aorta and coronary arteries. If an aortocoronary dissection occurs, routine CT follow‑up may be warranted, as it enables assessment of the aortic involvement extent. As previously noted, the Dunning classification correlates with prognosis: class I indicates limited and mild dissection, whereas class III reflects extensive and severe aortic involvement. A contrast-enhanced CT revealed that contrast was within the sinus of Valsalva, and no further surgical procedure was needed. We did not perform coronary angiography in the chronic phase because the procedure is invasive, and we sought to avoid unnecessary complications. After PCI, the patient received aspirin, rivaroxaban, and clopidogrel. Although dual antiplatelet therapy is generally required after deployment of a drug‑eluting stent, aspirin should be discontinued after two weeks when oral anticoagulation is administered to reduce the bleeding risk [12]. In accordance with the guideline recommendations, aspirin was discontinued two weeks after PCI.

Guide extension catheters are convenient and widely used when treating complex cases of coronary artery disease; clinicians should be aware of the associated rare complications. The lack of CAG assessment in both the chronic phase and the long‑term follow‑up represents a limitation. Furthermore, this study primarily evaluated anatomical findings, and functional assessment was not sufficiently examined. Examination of more cases is needed to elucidate the incidence, predictors, and prognosis of this rare complication.

## Conclusions

We encountered a case of iatrogenic aortocoronary dissection induced by the guide extension catheter. Advancement of the device can cause mechanical intimal injury when the catheter tip engages or tracks along the vessel wall. Once the intima is compromised, even a routine contrast injection may propagate the dissection. The mechanisms underlying these dissections are multifactorial, involving vessel anatomy, catheter configuration, support requirements, hemodynamic conditions, and operator maneuvers. Contrast injection through a guide extension catheter at the site of injury may further aggravate intimal disruption. Contrast staining in the sinus of Valsalva, sudden vascular haziness, and pressure damping should be regarded as critical warning signs. Therefore, once this complication is suspected, further contrast administration is contraindicated. The prognosis depends largely on the patient’s hemodynamic stability, the availability of IVUS, and the operator’s technical expertise. In this case, we assessed the injured coronary segment using IVUS and subsequently implanted a stent because the patient remained hemodynamically stable. Follow‑up CT imaging in the chronic phase demonstrated complete dissection healing. Operators should maintain a high level of vigilance when using guide extension catheters. Early recognition of intimal injury and prompt implementation of an appropriate bailout strategy are essential for successful management of this complication.
